# Adverse Event Burden, Resource Use, and Costs Associated with Immunosuppressant Medications for the Treatment of Systemic Lupus Erythematosus: A Systematic Literature Review

**DOI:** 10.1155/2013/347520

**Published:** 2013-04-03

**Authors:** A. Oglesby, A. J. Shaul, T. Pokora, C. Paramore, L. Cragin, G. Dennis, S. Narayanan, A. Weinstein

**Affiliations:** ^1^GlaxoSmithKline, U.S. Health Outcomes, 5 Moore Drive, Research Triangle Park, NC 27709, USA; ^2^United BioSource Corporation, 7101 Wisconsin Avenue Suite 600, Bethesda, MD 20814, USA; ^3^Human Genome Sciences, Medical Affairs, 14200 Shady Grove Road, Rockville, MD 20850, USA; ^4^Washington Hospital Center, Division of Rheumatology, 110 Irving Street NW, Washington, DC 20010, USA

## Abstract

This paper assessed the burden of adverse events (AEs) associated with azathioprine (AZA), cyclophosphamide (CYC), mycophenolate mofetil (MMF), methotrexate (MTX), and cyclosporine (CsA) in patients with systemic lupus erythematosus (SLE). Thirty-eight publications were included. Incidence of AEs ranged from 42.8% to 97.3%. Common AEs included infections (2.4–77%), gastrointestinal AEs (3.2–66.7%), and amenorrhea and/or ovarian complications (0–71%). More hematological cytopenias were associated with AZA (14 episodes) than MMF (2 episodes). CYC was associated with more infections than MMF (40–77% versus 12.5–32%, resp.) or AZA (17–77% versus 11–29%, resp.). Rates of hospitalized infections were similar between MMF and AZA patients, but higher for those taking CYC. There were more gynecological toxicities with CYC than MMF (32–36% versus 3.6–6%, resp.) or AZA (32–71% versus 8–18%, resp.). Discontinuation rates due to AEs were 0–44.4% across these medications. In summary, the incidence of AEs associated with SLE immunosuppressants was consistently high as reported in the literature; discontinuations due to these AEs were similar across treatments. Studies on the economic impact of these AEs were sparse and warrant further study. This paper highlights the need for more treatment options with better safety profiles.

## 1. Introduction 

Systemic lupus erythematosus (SLE) is a chronic systemic inflammatory autoimmune disease that can affect almost any organ and can present with musculoskeletal, neuropsychiatric, renal, cutaneous, and hematologic manifestations alone or in combination [1–3].

The treatment plan used to manage SLE is dependent upon the severity of the disease and organ systems involved. Antimalarials, nonsteroidal anti-inflammatory drugs (NSAIDs), and low-dose corticosteroids are used to treat mild-to-moderate disease, whereas higher doses of steroids are often used when symptoms remain uncontrolled [4]. Although not approved by the Food and Drug Administration for use in patients with SLE, immunosuppressants (e.g., azathioprine (AZA), cyclophosphamide (CYC), mycophenolate mofetil [MMF], methotrexate (MTX), or cyclosporine (CsA)) are prescribed to patients with moderate-to-severe symptoms to reduce steroid exposure in some patients [4–6]. Based on a clinical trial conducted in North America and Europe, it is estimated that more than half of patients with active SLE receive immunosuppressants as part of standard therapy [7]. However, immunosuppressant drugs are associated with significant short- and long-term side effects and require monitoring to assure their safe use [8]. 

Several systematic literature reviews have evaluated the incidence of adverse events (AEs) associated with immunosuppressants use in patients with SLE, but many focus only on MMF and CYC [9–16]. There have been no published systematic literature reviews that have evaluated resource use or costs associated with side effects of immunosuppressants in patients with SLE. To assess the burden of side effects associated with the off-label use of immunosuppressants in patients with SLE, a systematic review was conducted related to the incidence of AEs, discontinuation due to AEs, and the cost and resource use associated with AEs from five immunosuppressants of interest, including AZA, CYC, MMF, MTX, and CsA, in patients with SLE. 

## 2. Methods

A systematic review of English-language, EMBASE-indexed literature published between January 1980 and September 2011 was conducted using search terms associated with the five immunosuppressants of interest, SLE, AEs, discontinuation, resource use and costs. Supplemental targeted searches (i.e., nonsystematic searches) of published and non-published materials (e.g., health technology assessments and treatment guidelines) were also conducted to inform the review, including reviewing the Cochrane Collaboration. The bibliographies of review articles identified by the searches were further evaluated to trace any additional studies previously noted as relevant by prior reviewers. 

Abstracts were evaluated by one reviewer for inclusion according to predefined inclusion and exclusion criteria. Potentially relevant studies were retrieved in full and assessed for eligibility by two independent reviewers to provide consensus on the status of an article. Studies were included if they evaluated adult patients (≥18 years old) diagnosed with SLE with or without lupus nephritis receiving treatment with one of the five interventions of interest (AZA, CYC, MMF, MTX, and CsA) and reported one of the outcomes of interest. Letters to the editor, case reports, case series, nonsystematic reviews, studies of fewer than 50 patients, and articles without abstracts were excluded. 

Data extraction of relevant studies was conducted by one investigator and independently checked by a second reviewer. Point estimates for the percentage of patients experiencing side effects while receiving any of the interventions of interest was extracted directly from the text and tables of studies, where available. For some studies, this percentage was manually calculated. In view of differences in study design, these estimates were reported by study type for more straightforward comparison.

## 3. Results

**Figure 1:**
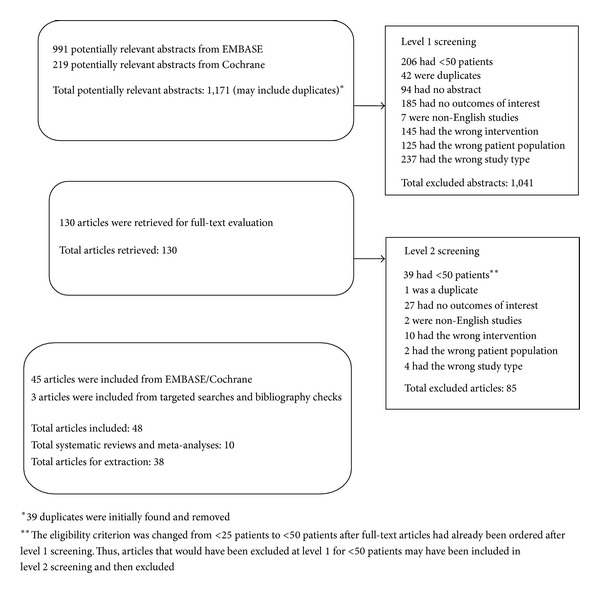
Identification and selection of full-text articles for review.

This systematic literature review yielded 1,171 abstracts, 130 of which were selected for further review (Figure 1). After eligibility assessment, 48 publications were included. Ten of these were systematic literature reviews or meta-analyses, and 38 were nonreview articles that were extracted for information related to the burden of AEs associated with immunosuppressants (34 articles), discontinuation due to AEs (21 articles) or the resource use (7 articles) or cost associated with AEs (0 articles). (Some studies reported more than one outcome of interest and were therefore counted under more than one category.) 

Of the 38 nonreview articles, 16 were randomized controlled trials (RCTs) and 22 were observational studies. After accounting for multiple publications from the same study, there were a total of 14 primary RCTs. Of the 14 RCTs, eleven were long-term studies evaluating patients who received immunosuppressants for 12 months or more, usually classified as maintenance treatment. Three RCTs were short-term studies, which evaluated patients receiving six-month induction therapy with one of the five immunosuppressants of interest. Across all studies, the mean study population size was 109 patients (range: 35–370 patients). Twenty-two studies evaluated only patients with lupus nephritis, while 14 studies assessed patients with any type of SLE. Treatment durations were similar in RCTs and observational studies, with reported durations from most studies ranging from 6 to 84 months and 6 to 43 months, respectively, and averaging 29 months and 17 months, respectively. Follow-up durations ranged more widely among RCTs (12 to 132 months) than observational studies (14 to 65 months), although average follow-up durations were similar in RCTs (57 months) and observational studies (48 months). Frequently reported doses for the interventions of interest ranged from 0.5 to 3 g/day (MMF), 0.5 to 4 mg/kg/day (oral CYC), 0.5 g/m^2^ every three months to 1 g/m^2^ monthly (intravenous CYC [IVC]), 0.5 to 4 mg/kg/day (AZA), 7.5 to 25 mg/week (MTX), and 1.5 mg/kg/day (CsA). 

### 3.1. Incidence of AEs

The majority of nonreview articles identified (34 of 38: studies included in this count reported information related to the incidence of AEs associated with the immunosuppressant drug of interest; some studies only reported discontinuation due to AEs and were therefore not included in this count, but they were included in the count of studies related to discontinuation.) examined the incidence of AEs associated with immunosuppressants (MMF, 10 studies; MTX, 1 study; AZA, 10 studies; CYC, 23 studies; CsA 3 studies) in patients with SLE. (Chan et al. 2005 was included as an RCT in the counts for both CYC and AZA; it examined CYC plus prednisolone for 12 months as induction therapy, followed by AZA for at least another year.) Table 1 summarizes the most commonly reported side effects for the five interventions of interest in RCTs and observational studies.

#### 3.1.1. MMF

Ten studies reported toxicities associated with MMF in patients with SLE [17–28]. (Information for some studies was published in multiple references; therefore, throughout this paper the number of citations may not match the specified count.) Overall, the incidence of AEs was high, ranging from 42.8 to 66.7% in observational studies and from 96.2 to 97.3% in RCTs evaluating therapy with MMF; serious AEs were 3.7% and 27.7%, respectively [17, 24, 26, 27]. Frequently observed AEs in patients taking MMF included infections (12.5–68.5% in RCTs; 3.9–44.4% in observational studies), while hematological toxicities (0–21.7% in RCTs; 0.5–5.6% in observational studies), amenorrhea (0–6.6% in RCTs), and alopecia (0–10.9% in RCTs) were also reported in these patients [17–28].

GI AEs frequently occurred in patients receiving MMF, with 9.1–61.4% of patients in RCTs, and 4.2–38.9% of patients in observational studies reporting a GI toxicity [17–25, 27]. Deaths occurred in 1.9% to 5.0% of patients taking MMF, but the studies did not report if these deaths were treatment or disease related [17–25, 28].

#### 3.1.2. MTX

Only one study evaluated side effects in patients with SLE taking MTX (route of administration not specified) [29]. While this short-term RCT reported a high incidence of overall AEs (93.0%), this rate was not significantly different from placebo (87%) [29]. Regarding specific side effects, the MTX group had a higher risk of GI (56.1% versus 33.3%; *P* = 0.05) and psychological side effects, such as mood disorder, (9.8% versus 0%; *P* = 0.05) when compared with placebo. 

#### 3.1.3. AZA

Ten studies reported AEs associated with AZA in patients with SLE [18–20, 23, 30–36]. Frequently reported side effects related to AZA were infections, leucopenia, amenorrhea, and premature ovarian failure [18–20, 23, 31–36]. In RCTs, infections were reported in 2.4–42.4% of patients, while hematological toxicities were reported in 6% to 50% of patients, and ovarian complications in 8% to 36% of patients [18–20, 23, 31–35]. Side effect rates were lower in observational studies with only 1.4% to 5.6% of patients reporting ovarian complications [36]. Deaths were reported in 0 to 25% of patients receiving AZA; however, it was not reported if the deaths were related to the study drug [19, 20, 23, 31, 32].

#### 3.1.4. CYC

Twenty-three studies observed CYC-associated toxicities, including oral CYC (8 studies) and/or IVC (21 studies) [17–20, 22, 24, 28, 30–32, 36–50]. Overall, the percent of patients experiencing AEs while taking IVC was 95% in RCTs and ranged between 57.5% and 65% in observational studies [17, 24, 49, 50]. Side effects reported included hematological toxicities (oral CYC: 7–25.8%; IVC: 1.4–38.7%), and hypertension (oral CYC: 3.2%; IVC: 2.5–50%) [17–20, 22, 24, 28, 31, 37, 39, 44, 46, 48, 50]. 

For patients receiving IVC, infections were often reported ranging from 11.8% to 77% in RCTs and 12.5% to 67.9% in observational studies [17–20, 24, 28, 31, 32, 37–39, 42, 44, 49, 50]. GI AEs were also commonly reported for patients receiving IVC with 29.4% to 66.7% of patients experiencing them in RCTs, and 18% to 58.8% in observational studies [17, 19, 20, 24, 37, 44, 46, 50].

The proportion of patients with infection while receiving oral CYC was high in both RCTs (33–40%) and observational studies (26–61%) [17–20, 22, 24, 28, 31, 32, 37–40, 42, 44–46, 48–50]. Further, in patients taking IVC, ovarian complications appeared in 1.9–58%, while in patients taking oral CYC, these toxicities were experienced by 28–71% [17–20, 22, 24, 30, 31, 36–41, 43, 44, 46–49].

While death rates in patients taking IVC (2.7–20%) [17, 19, 20, 22–24, 28, 31, 38, 40, 49] and oral CYC (6.5–22.2%) [31, 32, 45, 48] were similar, it was not reported if the deaths were treatment related [17, 22, 24, 28, 32, 37–39, 41, 48, 49].

In studies comparing the route of administration, GI AEs were more frequently observed in patients taking IVC (18%) than in those receiving oral CYC (7%), while alopecia (IVC: 23%; oral CYC: 31%), premature ovarian failure (IVC: 13–45%; oral CYC: 28–71%), and transient amenorrhea (IVC: 20%; oral CYC: 37%, *P* = 0.01) were more often reported in patients taking oral CYC than in those taking IVC [31, 44]. 

#### 3.1.5. CsA

Three studies reported side effects related to CsA treatment in patients with SLE [33, 34, 51]. In one observational study, 62.5% of patients reported experiencing an AE [51]. Frequently reported AEs included infections (6.4%–19.4%) and GI toxicities (3.9%–30.6%) [33, 34, 51]. Other toxicities reported in patients taking CsA included leucopenia (11.1–19.1%) and anemia (13.9–38.3%) [33, 34]. Hypertension was reported in 19.4–48.9% of patients with SLE taking CsA in RCTs [33, 34]. Further, increased creatinine occurred in 11.7–12.8% of patients with SLE taking CsA [33, 51]. Deaths occurred in 4.3% of patients, but these were not attributable to study drug [33].

#### 3.1.6. Direct Comparisons of Immunosuppressants

Ten studies directly compared at least two of the immunosuppressants of interest [17–20, 22–24, 28, 31–34]. CYC was associated with more infections than MMF (40–77% versus 12.5–32%, resp.) or AZA (17–77% versus 11–29%, resp.), and it was also related to more gynecological toxicities than MMF (32–36% versus 3.6–6%, resp.) or AZA (32–71% versus 8–18%, resp.) [17–20, 22, 24, 31, 52]. Specifically, in one short-term RCT, compared with CYC, MMF was associated with fewer infections (CYC: 40%; MMF: 12.5%; *P* = 0.0013), infections that caused hospitalization (CYC: 30.0%; MMF: 6.3%; *P* = 0.014), and amenorrhea (CYC: 36%; MMF: 3.6%;  *P* = 0.004) [18]. A short-term RCT reported that pyrogenic infections were significantly less frequent among patients receiving MMF than among IVC (relative risk: 0.36; *P* = 0.030) [22]. Further, a long-term RCT comparing AZA, MMF, and IVC found that amenorrhea (MMF: 6%; AZA: 8%; IVC: 32%; *P* = 0.03 for MMF versus IVC; *P* = 0.03 for AZA versus IVC) and infection (MMF: 32%; AZA: 29%; IVC: 77%; *P* < 0.005 for MMF versus IVC; *P* < 0.002 for AZA versus IVC) were lower in the AZA and MMF groups compared with the IVC group [19, 20]. Additionally, compared with IVC and oral CYC, AZA monotherapy was associated with significantly fewer herpes zoster infections (IVC: 25%; oral CYC: 33%; AZA: 11%; *P* < 0.05 for both comparisons) and premature ovarian failure (IVC: 45%; oral CYC: 71%; AZA: 18%; *P* < 0.01 for both comparisons) in another short-term controlled trial [31]. Further, AZA resulted in significantly fewer hemorrhagic cystitis events than oral CYC (0% versus 17%, resp.; *P* < 0.01) [31]. Finally, patients taking AZA experienced significantly more hematological cytopenias than MMF (14 episodes versus 2 episodes, resp.; *P* = 0.03) [23]. 

### 3.2. Discontinuation due to AEs

This systematic literature review yielded 22 studies reporting discontinuation rates due to side effects in patients with SLE taking MMF, MTX, AZA, CYC, or CsA (Table 2) [17, 18, 22–27, 29, 32–36, 38, 39, 45, 46, 50, 51, 53, 54]. Overall, these rates were generally similar across treatments. Seven studies evaluated MMF, and discontinuation rates due to toxicities in these studies ranged from 0% to 16.7% [17, 18, 22–27]. Eight studies analyzed AZA, reporting rates from 2.3% to 21.8% across study types [18, 23, 32–35, 39, 54]. Only three studies reported discontinuation rates due to AEs in patients taking MTX, with a range of 8.3% to 12.2% [29, 53, 54]. 

Eleven studies [17, 18, 22, 24, 32, 36–38, 45, 46, 50, 54] reported discontinuation rates due to side effects for patients taking CYC. In RCTs, 2.9–7.7% of patients discontinued IVC [17, 22, 24, 37, 38, 46, 50]. For oral administration, these rates were higher (9.7–44.4%) [18, 32]. Finally, three studies reported withdrawal rates due to AEs in patients with SLE taking CsA, which varied between 13.9% and 17.0% [33, 34, 51]. 

### 3.3. Resource Use and Cost

Eight studies reported resource use associated with MMF, AZA, MTX, CYC, or CsA in patients with SLE [18–20, 22, 27, 39, 40, 44, 55]. Generally, these studies reported resource use in terms of hospitalizations due to AEs; however, frequency measures used to report hospitalization varied among the studies resulting in an inability to make comparisons between studies. There were no studies in this review that evaluated costs or resource use associated with treatment of AEs on an outpatient basis or, in general, described costs associated with treating or monitoring AEs associated with immunosuppressants in patients with SLE. 

Three studies directly compared at least two interventions of interest [18–20, 22]. In a long-term RCT, compared with MMF, sequential therapy with CYC and AZA resulted in significantly more infections requiring hospitalization (6.3% and 30%, resp., *P* = 0.014) [18]. Another long-term RCT comparing hospitalization rates in patients receiving IVC, AZA, or MMF reported that patients receiving IVC (10 days per patient-year) were significantly more likely to require hospitalization for AEs than patients receiving either AZA (1 hospital day per patient-year; *P* = 0.03) or MMF (1 hospital day per patient-year; *P* = 0.007) [19, 20]. Further, a short-term RCT reported that five patients (6.7%) receiving IVC required hospitalization for vomiting and dehydration; no patients were reported in the MMF group to require hospitalization [22]. 

The remaining studies reported information on one intervention of interest. One observational study compared hospitalization rates due to infection in patients receiving immunosuppressants versus those receiving prednisone and reported no difference between the groups [55]. Notably, route of administration did not seem to affect rates of infection requiring hospitalization as exhibited in one observational study which found no significant difference between oral CYC (17%) and IVC (21%) (*P* = 0.50) [44]. A long-term RCT comparing doses of IVC reported that 22.2% of patients receiving high-dose IVC and 11.4% of patients receiving low-dose IVC experienced infection requiring hospitalization; the cumulative probability of severe infection was not significantly different between the groups (*P* = 0.20) [39]. Additionally, in one long-term RCT, at least three patients receiving CYC experienced infections requiring hospitalization; however, the article did not report enough information to estimate the risk of hospitalization due to infection [40]. Finally, in one retrospective study of 54 SLE patients receiving MMF, only one patient was hospitalized [27].

## 4. Discussion

This systematic literature review synthesized the burden of AEs associated with immunosuppressant therapies in patients with SLE. Overall, MMF, AZA, MTX, CYC, and CsA are associated with a high incidence of AEs including infections, hematological toxicities, GI events, and ovarian toxicities. Despite the availability of primary research on the incidence of AEs associated with immunosuppressants, the data are difficult to compare across studies in view of differences in study design. Discontinuation rates for these treatments have been observed between 0% and 44.4% [18–20, 23–27, 29, 32–34, 37, 46, 50, 51, 54]. 

Our systematic review has revealed several limitations in the current body of knowledge. For example, few studies evaluate the side effects of either MTX or CsA in patients with SLE and studies generally do not clearly distinguish between treatment-related AEs and those associated with SLE. Further, costs of managing side effects associated with immunosuppressants in patients with SLE are essentially nonexistent in the literature. Such studies are needed to better characterize the clinical and economic burdens of SLE immunosuppressant therapy to better understand the overall value of such therapy.

Several systematic literature reviews and meta-analyses comparing the incidence of AEs associated with immunosuppressants in SLE, mainly MMF and CYC, have been published, but with varying conclusions [9–12, 56–58] about treatment safety profiles. Specifically, systematic literature reviews have drawn different conclusions regarding the relative frequency of leucopenia, alopecia, and amenorrhea in patients taking MMF or CYC [10–12]. Findings from this systematic literature review indicate that MMF was associated with lower rates of gynecological toxicities and infection than CYC, but the statistical significance of this difference has been questioned in some studies [17–20, 22, 24, 31]. Lee et al. observed similar safety profiles related to MMF and AZA for maintenance therapy [11], which was consistent with the findings of this literature review as well as with those from a recently published RCT [17, 18, 22, 24, 31, 59]. However, one study in this review found AZA to be associated with more hematological cytopenias than MMF, and a newly published RCT found that significantly more patients discontinued treatment due to AEs when taking AZA than when receiving MMF [23, 59]. 

Few systematic reviews assessed incidence of hospitalization. Both Lee et al. and Moore and Derry found that the incidence of hospitalization associated with AEs was lower in patients with lupus nephritis taking MMF than in those taking CYC [9, 11]. The present systematic review also came to this conclusion. Because resource use was generally reported as hospitalizations due to AEs, and frequency measures used to report hospitalization varied among the studies, there was an inability to make comparisons between studies. Therefore, conclusions were drawn based on studies directly comparing the drugs of interest. For example, one study reports a single hospital day per patient-year for patients receiving MMF compared with ten for patients taking CYC [19, 20]. Further, Moore et al. observed that discontinuation due to AEs was not significantly different between patients receiving MMF and CYC [9]. While this outcome was not statistically compared in the studies in our review, this finding is consistent with the comparisons of the results among the included studies. 

This systematic literature review is unique from previously published systematic literature reviews. For example, one of the objectives of this review was to identify studies evaluating resource use and costs associated with side effects of CYC, AZA, MMF, MTX and CsA in patients with SLE. Further, this review included studies of SLE patients of all subtypes, whereas the majority of previously published reviews focused on lupus nephritis patients only. This study also compares the literature associated with five immunosuppressants instead of only MMF and CYC. Additionally, the review included immunosuppressants acting as both induction and maintenance therapies instead of focusing only induction therapy. 

This study has some limitations. First, a systematic literature review is limited by the effectiveness of its predefined search strategy (e.g., search terms, databases used, inclusion/exclusion criteria, etc) to identify all relevant articles on the topic of interest. Second, the data are difficult to compare across studies due to the diversity in study methodology, including dosing regimens, study and follow-up duration, and the varying concomitant therapeutic regimens. Further study is warranted to assess the potential for using meta-analysis to quantitatively synthesize the burden of AEs associated with each immunosuppressant of interest and provide more precise incidence estimates. Another limitation in this study comes from the short duration of many of the studies included in this review. Because long-term use of immunosuppressants may be associated with increased oncogenicity [60], the risk of malignancy may be underreported herein. Finally, this study does not take into account the role of corticosteroids in the predisposition to infection in SLE [61, 62]. Thus, the attribution of infection to one or another immunosuppressive agent could as well be related to the concomitant use of oral or parenteral corticosteroids. 

In summary, this paper provides a comprehensive overview of the side effects associated with immunosuppressant medications that are used in the management of SLE. Since immunosuppressants are used to treat SLE patients who are not optimally controlled on other forms of therapy and because SLE is a chronic disease with persistent inflammatory disease activity, this paper further highlights the need for treatment options with better long-term safety profiles. Biologics, which have more targeted mechanisms of action, are now being used to treat immune-mediated disorders, such as SLE, and, in theory, use of these targeted agents may result in fewer drug-related adverse events than conventional immunosuppressants; however, the cost implications of using these treatments for SLE remain unknown [63, 64]. Belimumab is the first biologic therapy introduced for SLE, but has not been used for a sufficient period of time to calculate a cost-benefit analysis as has been done with biologics for some other conditions [65].

## Figures and Tables

**Table 1:** Summary of commonly reported AEs by intervention.

	At least 1 AE	Infections	GI	Amenorrhea	Hematological	Death
				and/or ovarian complications		
MMF						
RCTs (*n* = 5)	96.2–97.3%	12.5–68.5%	9.1–61.4%	0–6%	0–21.7%	1.9–5.0%
Observational (*n* = 5)	42.8–66.7%	3.85–44.4%	4.2–38.9%	N/A	0.5–5.6%	N/A
MTX						
RCTs (*n* = 1)	93.0%	4.9%	56.1%	N/A	26.8%	N/A
Observational (*n* = 0)	N/A	N/A	N/A	N/A	N/A	N/A
AZA						
RCTs (*n* = 7)	N/A	2.4–42.4%	3.2–21.4%	8–36%	6–50%	0–25%
Observational (*n* = 3)	N/A	N/A	1.3%	1.4–5.6%	16.7%	N/A
IVC						
RCTs (*n* = 8)	95%	11.8–77%	29.4–66.7%	2.2–56.3%	1.4–38.7%	2.7–20%
Observational (*n* = 13)	57.5–65%	12.5–67.9%	18–58.8%	1.9–58%	2.5–7.7%	3.0–20%
Oral CYC						
RCTs (*n* = 3)	N/A	33–40%	3.2%	36–71%	25.8%	6.5–22.2%
Observational (*n* = 5)	N/A	26–61%	7%	28–37%	7%	N/A
CsA						
RCTs (*n* = 2)	N/A	6.4–19.4%	17.0–30.6%	N/A	11.1–38.3%	4.3%
Observational (*n* = 1)	62.5%	N/A	3.9%	N/A	N/A	N/A

AZA: azathioprine; CsA: cyclosporine; CYC: cyclophosphamide; IVC: intravenous cyclophosphamide; MMF: mycophenolate mofetil; MTX: methotrexate; N/A: not available; RCT: randomized controlled trial.

**Table 2:** Discontinuation rates by intervention and study type.

	MMF	MTX	AZA	IVC	Oral CYC	CsA
RCTs (*n* = 11)	1.4–13%	12.2%	2.3–20%	2.9–7.7%	9.7–44.4%	13.9–17.0%
Observational (*n* = 11)	0–16.7%	8.3–11%	19–21.8%	1.9–18.8%	5.6%	16.1%

AZA: azathioprine; CsA: cyclosporine; CYC: cyclophosphamide; IVC: intravenous cyclophosphamide; MMF: mycophenolate mofetil; MTX: methotrexate; N/A: not available; RCT: randomized controlled trial.
